# Improving Brain Tumor Research in Resource-Limited Countries: A Review of the Literature Focusing on West Africa

**DOI:** 10.7759/cureus.372

**Published:** 2015-11-03

**Authors:** Saidu I Ngulde, Francis Fezeu, Arjun Ramesh, Shayan Moosa, Benjamin Purow, Beatrice Lopez, David Schiff, Isa M Hussaini, Umar K Sandabe

**Affiliations:** 1 Department of Microbiology, Immunology and Cancer Biology, University of Virginia Health System, Charlottesville, Virginia; 2 Department of Veterinary Physiology, Pharmacology and Biochemistry, University of Maiduguri, Nigeria; 3 Department of Neurosurgery, University of Virginia Health System, Charlottesville, Virginia; 4 Department of Neurology, University of Virginia Health System, Charlottesville, Virginia; 5 Department of Pathology, University of Virginia Health System, Charlottesville, Virginia; 6 Department of Pharmacology and Toxicology, University of Maiduguri, Nigeria

**Keywords:** brain tumor, west africa, resource-limited countries

## Abstract

Neoplasms of the brain are often overlooked in resource-limited countries. Our literature search via AJOL and PubMed demonstrated that brain tumor research is still a rarity in these regions. We highlight the current status, importance, challenges, and methods of improving brain tumor research in West Africa. We suggest that more attention be given to basic, clinical, and epidemiological brain tumor research by national governments, private organizations, international organizations, non-governmental organizations (NGOs), and individuals in this region.

## Introduction and background

Globally, death from cancer alone is comparable to the combined deaths from HIV/AIDS, tuberculosis, and malaria. Cancer was originally believed to be a disease of developed nations, but recent estimates indicate that it is also a major health burden in resource-limited countries. Although benign and malignant brain tumors typically have a different prognosis, they are typically part of a neurooncology program. Therefore, the term 'brain tumor' is used, for practical reasons, interchangeably with brain cancer in this review in order to easily describe the lack of neurooncological facilities. Fifty-three percent of the estimated 12.7 million new cancer cases in 2008 were from developing countries, and the yearly incidence is expected to rise to 27 million by the year 2050 [1-2]. It is becoming more obvious that oncological diseases are no longer a concern only of the developed world, as low-income countries also have an increasing burden of tumors. The challenge of treating cancer in low-income countries is worse than in developed nations due to a lack of proper healthcare organization [2-3].

Generally, there is far less cancer research in resource-limited countries compared to developed countries. Nevertheless, there are a few research centers, as well as public and private organizations, that partner on research in breast, cervical, prostate, and other types of cancers. Notable examples include collaborations between Roswell Park Cancer Institute (RPCI)/State University of Buffalo, New York with two Nigerian hospitals: the University of Nigeria Teaching Hospital (UNTH) in 2009 and the Lagos State University Teaching (LASUTH)/College of Medicine in 2013. Also, in 2012, five different organizations with an interest in the research and control of cancer in Africa met in London to deliberate on the best way forward [4-5].

Brain tumors have largely been underestimated and ignored in resource-limited countries, and these types of tumors are not even listed among the important cancers in West Africa, which has had a serious negative impact on brain tumor research in this region. With this perspective, we highlight the status, importance, challenges, and methods of improving brain tumor research in resource-limited countries with an emphasis on West Africa. This was performed by systematic review of the literature from PubMed and the African Journal Online (AJOL). The following search terms were used: “brain, cancer, tumor, West, Africa”. A Google search of the internet was also conducted to find additional information.

## Review

### Epidemiology of brain tumor in resource-limited countries

The world annual incidence of primary brain tumors has been estimated to be 3.7 and 2.6 for every 100,000 men and women, respectively, with a higher rate in developed than developing countries. The mortality rates follow a similar pattern across sex and economic development [2, 6]. In Nigeria, like in many West African countries, little research has been conducted on the incidence of brain tumors. In a hospital-based cancer registry (HBCR) conducted in the 1990’s, brain tumor was ranked third in incidence at Jos University Teaching Hospital among all neoplasms, which was surprisingly greater than the incidence of breast, genitourinary, and lung cancers [7]. In another HBCR report from 2009-2010, incorporating data from 11 cancer registries in Nigeria, 6,484 cancer cases were recorded indicating prostate (29.2%) and colorectal (7.0%) cancers were the most common in males, while breast (40.3%) and cervical (17.3%) cancers were the most common in females [8]. However, 50% and 33% of the male and female cases, respectively, were not reported by type, with brain tumor being completely overlooked. At Lagos University Teaching Hospital, Nigeria, cancer death accounted for 4.7% of all causes of death with breast cancer having the highest cause (25.6%). CNS-associated cancer was responsible for 4.9% [9]. In Abidjan, 362 brain cancer cases were recorded from a single hospital within a period of 11 years while, in Nigeria, 210 intracranial neoplasms were confirmed from a single hospital within the same time period [10-11]. Another report from Southwest Nigeria indicated that brain cancer represented 3.9% of total cancers. It was the sixth most common neoplasm after breast (20.2%), cervical (7.9 %), fibroid (4.4%), liver (4.4%), and stomach (4.3%) neoplasms, while its prevalence was higher than that of pancreas (3.8%), prostate (3.3%), and lung (3.0%) neoplasms [12]. These are facts from HBCRs as opposed to population-based registries, which cover only 7.2% of the total population [13]. In Ghana, 30 neurological tumors were seen in one hospital within a period of two years, while 3.1% hydrocephalus cases in The Republic of Niger were found to be as a result of tumors [14-15].

The risk factors associated with brain tumors include radiation exposure, hereditary factors, age, sex, ethnicity, infections, and heavy metal exposure. In the West African context, high-dose radiation, age, infections, and heavy metals are important factors to consider. The application of radiation in the diagnosis and treatment of disease has increased in resource-limited countries in recent years, but these are with attendant increased risk of brain tumors. A study has shown that patients receiving dental radiography annually were at higher risk of developing meningioma than those receiving similar treatment in less than every fifth year [16]. It has also been shown that atomic bomb survivors have an increased risk of brain malignancies, and there are reports that exposure to even moderate doses of radiation is associated with high incidence of all central nervous system tumors [6, 17].

There is an increasing risk of brain tumor development with age, as with most neoplasms. There is an expected increase in the life expectancy in the resource-limited nations [18]. As the people of this region age and the population continues to grow, the incidence of brain tumors will also increase. According to the World Bank population database, the population in sub-Saharan Africa has been growing steadily at a rate of between 2.6% and 2.9% annually. Since the 1980’s, the percentage of the population above the age of 65 has increased from 2.9% to 3.1%. This increase in both the geriatric population and overall population indicates that there are an increasing number of people at risk for brain tumors in sub-Saharan Africa [19].

Certain viruses, including the human papillomavirus and HIV, have been linked to certain types of cancers. With the high prevalence of HIV infection and AIDS in West Africa, there is the concern for a greater risk of HIV-associated malignancies in this population. In fact, due to increasing use of antiretroviral therapy throughout the world resulting in longer expected lifespans for HIV-infected patients, over 40% of these patients now go on to develop a malignancy [20-21]. Primary central nervous system lymphoma (PCL), an AIDS-defining illness that represents one type of non-Hodgkin lymphoma (NHL), is poorly understood, but is thought to occur due to the poor immune regulation of the Epstein-Barr virus and other viruses [22]. Ten percent of patients who are infected with HIV develop NHL [23], and patients diagnosed with AIDS have a 1,000-fold increased incidence of PCL [24]. Thus, it is imperative that any serious interventions in Africa to reduce the incidence of brain tumors incorporate methods of reducing HIV-infection rates, such as through public education initiatives and protected sex campaigns.

Technological improvements in resource-limited countries in recent years, such as improved sewage waste and water treatment and modernized farming, have led to an increase in environmental contaminants. Ingestion of these contaminants, such as chlorinated compounds, is linked to an increased risk of brain tumors [6, 25]. Cadmium and lead have been associated with cancer. Inorganic lead can cause direct damage to DNA, inhibit DNA synthesis and repair, and cause oxidative damage through free radical generation [26]. Thus, one of the long-term effects from increased lead ingestion is an increased risk of brain tumor. It has been reported that there was a positive association between lead exposure and development of meningioma [27-28]. The blood threshold level of lead is 5 μg/dl, and chelation therapy is required when the blood level is 45 μg/dl and above [29]. Recently, there was an outbreak of lead poisoning in gold mining areas in some parts of Nigeria affecting humans and animals in the locality. A study conducted in 2013 reported that 92.5% of children studied in the affected areas had a blood lead level above 5 μg/dl, while 11.1% of them had a blood lead level above 45 μg/dl. Fourteen percent of their households had a soil lead level above 400 mg/kg [30]. Soil lead levels greater than 100,000 ppm were found in some homes [31]. It was implicated that edible snails from waste dump sites in Nigeria were associated with increased risk of childhood lead consumption, often leading to ingestion of 0.12 mg/kg/day, which was higher than the WHO limit of 0.25 mg/kg/week [32-33]. Also, assessment of heavy metal levels in canned meat and fish showed lead levels (0.988 mg/kg in fish and 0.816 mg/kg in meat), significantly higher than the WHO limits of 0.5 mg/kg and 0.1 mg/kg, respectively [34].

### Strategy for improved brain tumor research in resource-limited West Africa

Awareness

It is important to increase the awareness of brain tumors among doctors and the general population in West Africa in order to improve the diagnostic accuracy and increase research funding. There should be increased suspicion for this disease in patients with a longstanding history of headaches, as well as in those with psychiatric problems, in order to prevent inappropriate admission to psychiatric hospitals [7]. Studies of the awareness of other types of cancer in West Africa showed varied levels of awareness within the society: 80%, 15%, 11.4%, and 67% [35-38]. However, records of the awareness of brain tumors in this region are rare. A call for periodic seminars and conferences in resource-limited countries will increase awareness.

The societies of resource-limited countries are often found to have a poor understanding of the pathophysiology of brain tumors. The attitude toward managing this disease needs to be changed in many of these societies. Over 70% of patients in resource-limited countries seek alternative sources of therapies from traditional medicine practitioners (TMP), with only the most advanced-stage cases ever reaching hospitals [39]. For example, in Ghana, the ratio of the population to TMP and medical physicians is 1:400 and 1:12,000, respectively [40]. The general belief in West Africa is that cancer is incurable and that orthodox medicine can aggravate the condition. In addition, many people in this region believe that cancer is caused by supernatural powers beyond the capacity of modern health care; thus, many sufferers of brain tumors choose to seek spiritual healing instead. Some of these people believe that piercing the body of a cancer patient with a metallic object, such as needles or blades, can result in metastasis. Also, patients with brain tumors who are affected psychologically are believed to be best managed traditionally [41-42]. All these beliefs result in the distancing of brain tumor sufferers from hospitals. Therefore, a logical first step in combating improper treatment is for governmental and non-governmental leaders and organizations in the area to promote health education. Insufficient population-based cancer registries have been blamed for the likely underestimated incidences of all cancer types in Africa [4, 43]. The International Network for Cancer Treatment and Research (INCTR) Cancer Registry Program, which was established in 2012 and is coordinating an African Cancer Registry Network (AFCRN), should not make the same mistakes as other studies in overlooking brain cancer in its surveillance [44]. Brain cancer patients rarely reach the modern health facility centers, and careful work will need to be done to add these people to the registry.

The use of media is another means of improving public awareness. For example, an enlightenment program on brain tumor can be organized and broadcast weekly or monthly on popular radio stations in local dialects that can be understood by the majority of the population. Governments or NGO’s can support such programs. Publicity via radio in resource-limited countries has a greater impact than other forms of media because it has wide coverage and is inexpensive; thus, almost every household owns a radio in the rural areas of West Africa [45]. In addition, radio is popular because it is portable and an audible tool, making it suitable for the predominantly illiterate population [46]. Enlightenment programs can also be arranged via community leaders. In Northern Nigeria, there is an organized system of community leaders that are well-respected in the region. Acting through these leaders will be important in promoting a positive change in the health awareness of their followers.

Planning and Training Programs

The number of trained personnel in the field of brain research in resource-limited countries is grossly inadequate. There are very few neurosurgeons in these countries. In Nigeria, with a population of 140 million, there were only between 9 and 30 neurosurgeons practicing according to different reports [47-49]. Increased awareness of brain tumors would lead to increased funding for research and recruitment of neurosurgeons. Through training neuroscientists and neurosurgeons, awareness will undoubtedly also increase within the society. Support for scientific training in West Africa is available in other clinical areas but lacking for brain research. There is a training grant for HIV-associated malignancies in sub-Saharan Africa supported by the National Institute of Health (NIH), Operation Stop Cervical Cancer in Nigeria (OSCCN), and the Exxon Mobil Foundation [4]; however, a call for similarly supported training programs for brain cancer research is needed. Training for primary healthcare providers, clinicians, administrators, and researchers are required in the field of brain cancer research, and the local, state, and federal governments should initiate, organize, and support it financially. There are already healthcare service delivery systems in resource-limited-countries, and a brain cancer initiative can be integrated into these existing plans where possible. At the grassroots level, primary healthcare providers should be trained to fully understand the significance of brain cancer and the need to refer brain cancer sufferers to the appropriate specialists. There is a handful of tertiary health institutions, universities, and research institutes in these countries, but they need more trained personnel in the field of brain cancer for diagnosis, treatment, control, and research. A call for the establishment of NGOs with the intention of voluntarily uniting the families or close associates of victims of brain tumor and other diseases of the brain will assist in bringing out the actual incidence of brain cancer, as well as to weaken some of the superstitions associated with brain cancer in West Africa.

Infrastructure

In resource-limited countries, there is a general lack of infrastructure. Thus, this is an important area where improvements can be made to have a positive impact on brain tumor research. Governmental and private organizations should invest in both improving the available infrastructure and generating new infrastructure. For example, a brain cancer research institute can be established in each country, which can serve as a focal point for research, coordination, and collaboration with other agencies both within and outside the country. Modern facilities for quality research should be provided in the existing relevant institutions of higher learning. There should be the creation of a brain cancer research department in each of these institutions. A well-developed infrastructure is also needed for proper diagnostic and management of patients at health institutions. For instance, as of the year 2012, there were less than ten magnetic resonance imaging (MRI) facilities in Nigeria, and the Ghanaian government was trying to acquire four new MRI facilities [50-51]. For Nigeria, with a population of over 150 million at that time, the number of facilities were grossly inadequate. The number of facilities for the documentation of cases, screening of patients, confirmation of diagnosis, treatment, control, and palliation are inadequate and need to be expanded. Drugs for treatment are often not accessible, and they are extremely expensive when they are [42]. Government subsidization of drugs will be necessary for controlling drug prices.

Collaboration

Resource-limited countries often have inadequate facilities and manpower to support brain tumor research. Through collaboration, most of these shortcomings can be overcome. There are several governmental and institutional collaborative activities between high-income and low-income countries, but this is insufficient in the area of brain tumor research. Notable examples include the Medical Education Partnership Initiative (MEPI), the Human Heredity and Health in Africa Initiative supported by the NIH and Wellcome Trust, the International Agency for Research on Cancer (IARC) focusing on liver, cervical, lung, and breast cancers, and the International Cancer Technology Transfer (ICRETT) fellowship providing scholarships for professional skill acquisition in research and clinics in Africa [4]. The partnership on non-communicable diseases between the University of Ibadan and the University of Chicago has resulted in the training of many personnel from the resource-limited region of Nigeria and has brought out new insight in the genetics of breast cancer. Such collaborations are welcomed and should be maintained and widened to include the field of brain tumors. The Multidisciplinary University Traditional Health Initiative, a European Union funded project with the primary objective of strengthening African Universities in herbal medicine research, is another example of collaboration between advanced nations and resource-limited countries. However, similar collaborations with an interest in brain tumors still need to be established. The objectives of these existing partnerships could be expanded to include brain tumors. This will serve as an avenue for training brain tumor researchers in resource-limited countries. International organizations, such as WHO, NIH, and the African Union would be ready to support initiatives that would clearly improve the health of the people in low-income countries. The African Organization for Research and Training in Cancer (AORTIC) partners with African countries to promote training and national and international collaborations with a focus on Africa [52]. However, there should also be clearly defined objectives from governments within the region. Collaborations in research should be encouraged within the institutions in low-income countries. Because of inadequate facilities and manpower, it is imperative that there is intra-institutional collaboration before meaningful research can be achieved.

Research

Despite the previously described challenges, there should be more of an interest in brain tumor research by West African scientists. Understanding the many aspects of brain cancers is challenging researchers across the world. Research efforts to treat or to significantly prolong the life of glioblastoma (GBM) patients have yet to provide a solution [53]. An African scientist could provide the much-needed solution to this disease that has yet to have a cure. Research in the epidemiology of the tumor will provide insight into the distribution of the disease in this part of the world. Genetic factors responsible for predisposing a particular race can be investigated. For example, a report on GBM in America indicated the five-year relative survival of Caucasians was 33.5%, whereas the survival rate in black Americans was 37.0% [6]. Research on the attitude and belief regarding brain tumors in West Africa can also be implemented in order to recommend strategies for improving hospital-based registration and treatment of brain tumor cases.

Africans have a strong belief in the power of their traditional drugs in the treatment of diseases such as cancer. These drugs are mostly made from local plant materials. Research of traditional medicine is highly advocated. Many drugs used today are from plant sources (e.g., the anticancer drugs vincristine, paclitaxel, and camptothecin) [54]. Therefore, efforts should be intensified in the research of African medicinal plants. Collaborative efforts between the traditional medicine practitioners (TMPs) and brain cancer researchers is needed in the West African sub-region to find new ways of promoting research on local herbs for the benefit of not only the region but the entire world. One major problem with the TMPs is the secrecy in their formulations and the fear of losing their share of patients if the ingredients of their medicines become known to the scientists [55]. With the intervention of government, NGOs, and community leaders, such collaborative approaches to benefit both sides can be achieved. The outcome of such research can go a long way in reviewing the significance of traditional medicine.

Funds

Funding is an essential part of sustained brain tumor research in West Africa; thus, there should be appropriate plans to raise funding for research. Governments at all levels, private organizations, international organizations, NGOs, and individuals should all be persuaded to help sponsor research. Private organizations, such as pharmaceutical companies, should be encouraged to sponsor brain tumor research in West Africa. They should extend their financial support to include brain tumors in resource-limited countries because of the increased cancer burden in these regions and the associated demands for their products. Local, state, and federal governments should specifically budget funds for brain tumor research. Usually, there are plans and budgets by the governments for cancer research, but the priorities for brain tumor research should be increased. Wealthy individuals, such as politicians, and especially those that have close relatives affected by the disease, would likely be willing to support brain tumor research. Research funding in West Africa by international organizations and institutions in advanced nations is available mainly through collaborations, but those with a focus on brain tumor research are rare. While the Global Fund to Fight AIDS, tuberculosis, and malaria is helpful to the region, this and other organizations should start thinking about cancer as it is clear its incidence is increasing in low-income countries. There is an active interest in brain cancer research; according to the NIH, there are at least 12 different research organizations dedicated to brain cancer research. Given that there are clear demographic differences in outcomes between the US population and the population in sub-Saharan Africa, it makes sense for some of these organizations to fund research in the region. The NIH has already set a precedent for other organizations, such as the Centers of AIDS Research Program for HIV research and the Ghana Prostate Cancer Study for prostate cancer. Given the demonstrated research interest in intracranial tumors, global disease burden, and the need for further research in these populations, the NIH or other agencies should begin to fund brain cancer research in Africa as well [19, 56].

## Conclusions

The burden of oncological pathologies in West Africa is rapidly increasing, but brain tumors are often overlooked. Brain tumor research should receive more attention in this region. A call for improved research in the region is needed in basic, clinical, and epidemiological research and should be supported by the government, private organizations, international organizations, NGOs, and individuals.
